# Embedding patient and public involvement: Managing tacit and explicit expectations

**DOI:** 10.1111/hex.12952

**Published:** 2019-09-20

**Authors:** Fiona Poland, Georgina Charlesworth, Phuong Leung, Linda Birt

**Affiliations:** ^1^ School of Health Sciences Faculty of Medicine and Health Sciences University of East Anglia Norwich UK; ^2^ Research Department of Clinical, Educational and Health Psychology University College London London UK; ^3^ Research and Development Department North East London NHS Foundation Trust London UK; ^4^ Division of Psychiatry University College London London UK

**Keywords:** co‐applicant, Constructionist, co‐research, dementia, Patient and public involvement, peer research

## Abstract

**Background:**

Evidencing well‐planned and implemented patient and public involvement (PPI) in a research project is increasingly required in funding bids and dissemination activities. There is a tacit expectation that involving people with experience of the condition under study will improve the integrity and quality of the research. This expectation remains largely unproblematized and unchallenged.

**Objective:**

To critically evaluate the implementation of PPI activity, including co‐research in a programme of research exploring ways to enhance the independence of people with dementia.

**Design:**

Using critical cases, we make visible and explicate theoretical and moral challenges of PPI.

**Results:**

Case 1 explores the challenges of undertaking multiple PPI roles in the same study making explicit different responsibilities of being a co‐applicant, PPI advisory member and a co‐researcher. Case 2 explores tensions which arose when working with carer co‐researchers during data collection; here the co‐researcher's wish to offer support and advice to research participants, a moral imperative, was in conflict with assumptions about the role of the objective interviewer. Case 3 defines and examines co‐research data coding and interpretation activities undertaken with people with dementia, reporting the theoretical outputs of the activity and questioning whether this was co‐researcher analysis or PPI validation.

**Conclusion:**

Patient and public involvement activity can empower individual PPI volunteers and improve relevance and quality of research but it is a complex activity which is socially constructed in flexible ways with variable outcomes. It cannot be assumed to be simple or universal panacea for increasing the relevance and accessibility of research to the public.

## 1 INTRODUCTION

Drawing on lived experiences from ‘experts by experience’ to enable patient and public involvement (PPI) is now expected and often reported as integral to ‘good’ research design. In applied health and social care research, funders may require PPI activity to be specified in applications and expect PPI to improve the quality and public relevance of research.^1^, ^2^ Public discourses of PPI activity present it as an activity that intrinsically empowers lay people to directly influence research questions and process.^3^ Yet, PPI involves complex interactions between people, whose differing reasons for doing it will shape their contributions.^4^ In dementia research, an individual's clinical dementia symptoms, alongside carers’ and researchers’ desires to ‘protect’ potentially vulnerable people, can limit active and meaningful involvement for the person with dementia.^5^

By taking a constructionist lens to this participatory methodology, we highlight the multifaceted nature of PPI and the potential challenges inherent in acknowledging and addressing all parties’ tacit and explicit expectations. We critically reflect on the practical processes and consequences of embedding three distinct forms of PPI into a five‐year grant‐funded programme Promoting Independance in Dementia (The PRIDE Study), involving research with people with dementia and their family carers. We use case studies to identify theoretical and moral challenges.

Patient and public involvement is rooted within the participatory research movement's calls to involve the public in research affecting them, placing value on partnership and collaboration.^6^, ^7^, ^8^ PPI activity occurs across a spectrum of participation types and at all research stages. PPI members may collaborate on project delivery, including providing advice on patient information sheets and other research material as advisory group or steering group members. However, PPI activity can now include the co‐applicant role on funding bids. Here PPI colleagues collaborate with professional researchers in developing and applying for research grants.^9^ An extended form of PPI activity is co‐ or peer‐researchers, where people with lived experience of the condition under study work alongside academic researchers.^10^, ^11^ Co‐research is relatively uncommon in dementia research. Challenges to involvement include preconceived ideas on acceptability of the activity.^12^Latterly, there has been increased interest in co‐research with publications reporting on processes for including people with dementia, although these often focus on the practicalities of organizing such activity.^14^, ^15^

A clear consistent definition of types of PPI activity remains elusive.^16^ This means lay members, researchers, monitoring bodies and funders may embark on PPI activities with differing tacit assumptions and expectations. Different expectations need to be negotiated for mutually effective ways of working. Given that shared understandings are constructed through social relations, we suggest PPI activity should be seen as a site of multiple misunderstanding, tensions and unmet expectations. This is especially so in dementia research where the voice of the person with dementia has historically been excluded from research.^17^, ^18^ For clarity, in this paper, we use the phrase ‘people with dementia’ to refer to the person with the diagnosis and use the term ‘carer’ to refer to supporting family members. We use the term PPI member or PPI person to discuss general PPI activity.

Increasingly, national and local PPI groups are providing platforms for people with dementia to articulate their experiences and influence research.^19^, ^20^ This reflects a consensus on the positive aspects of enabling people with dementia to be active citizens.^21^ However to avoid tokenism, the complexity of representation in PPI activity in dementia research needs acknowledging. Debates about representing voice and power‐holding within PPI activity play out at every level.^22^ Conceptual frameworks may ground PPI activity within ideological rights and values, which reflect on and redress power imbalances.^23^ Such a framework would compel activity that includes voices of people with dementia. However, more pragmatic outcome‐based frameworks^23^might foreground participants’ competency to relay information. This was seen historically when researchers included carers’ voices without acknowledging voices of people with dementia.

The impact of PPI activity on research quality, or on links between benefits and economic costs, is rarely reported.^24^, ^25^ More holistic knowledge on PPI activity would entail reporting the complexities of PPI activity in practice, including discussion on the distinctive theoretical and moral challenges experienced when undertaking co‐research in dementia research. Therefore, here we examine whether professional researchers’ and PPI members’ differing agendas create misunderstandings as well as shared understanding and whether this creates any opposing views and expectations and any potential for consensus.

### 1.1 Setting the scene

**Figure 1:**
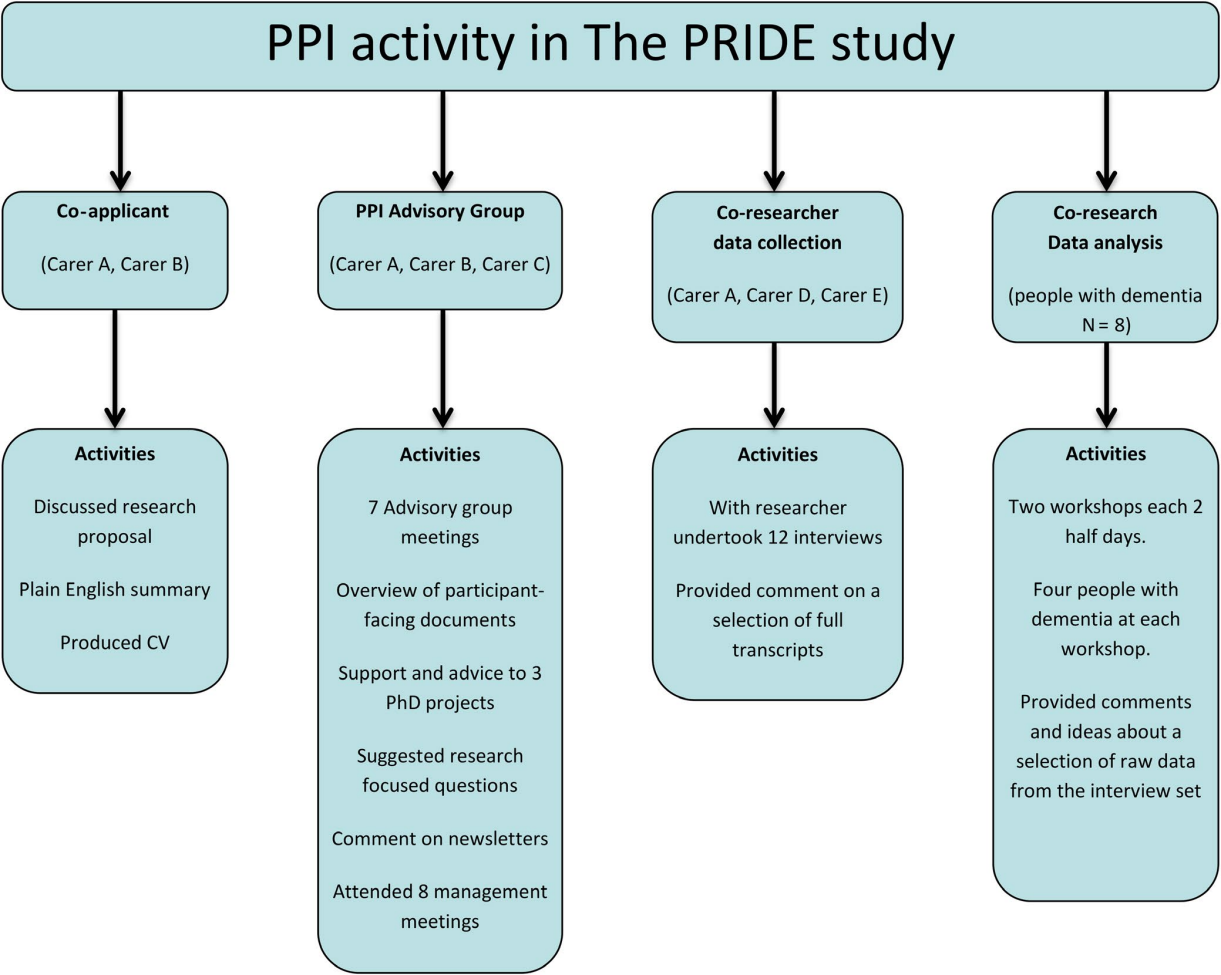
Types of patient and public involvement (PPI) activity in The PRIDE Study

Setting the context of PPI complies with best practice for reporting such involvement.^26^ The PRIDE Study recruited from four geographical areas of England. The work packages comprised of a quantitative exploration of factors affecting cognitive function; a qualitative exploration of experiences of cognitive impairment in older people; and development and feasibility testing of a technological intervention to enable people with dementia to remain independent.^27^Several distinct types of PPI were planned, including PPI advisory members and co‐research with people with dementia and carers (see Figure 1). All PPI activity was costed to finance six monthly PPI advisory group meetings; PPI volunteer attendance at management meetings; and co‐research activity. The three PPI advisory group members were all current or former carers of people with dementia, living in one recruitment area. Recruitment to co‐researcher roles occurred across sites, facilitated by gatekeepers: NHS mental health trusts, Alzheimer Society, dementia support groups and personal contacts. During data collection, we were unable to recruit people with dementia into co‐research roles. This was partly because health professionals and carers assumed the cognitive capabilities of the person with dementia would limit their potential contribution.^12^Carers also raised physical concerns: a person with dementia expressed an interest in the co‐interviewer role, but their relatives vetoed their involvement arguing their limited mobility restricted access to this activity. Three carers were recruited as co‐researchers; they undertook interviews and validated and expanded researchers’ analyses.

Critical case method studies a phenomenon in its action context.^28^ Using a critical case researchers can define the nature of an incident and its impact on project aims, thereby investigating the factors, behaviours and experiences of those people involved in a critical event.^29^, ^30^ We define a critical case here, as an identified set of assumptions and interactions which challenge any stated rhetoric of what PPI is and does. Each of our critical cases revealed tensions between actions and expectations, relating to limits of empowerment in PPI roles, benefits from PPI activities for PPI members and benefits of PPI co‐research to research quality. The critical cases are designated: Case 1 ‘Undertaking multiple roles—acknowledging differing responsibilities’; Case 2 ‘My experiences matter’—the complexity of co‐research interviewing’; and Case 3 ‘Co‐research or validation—doing data analysis with people with dementia?’. Critical case methods can be used to generate analytical generalizations^31^ enabling features of these cases, arising in a research project involving people with dementia and carers, to be transferred to other PPI contexts. We draw on empirical literature to set our reflections within a wider context.

## 2 CRITICAL CASES

### 2.1 Case 1: ‘Undertaking multiple roles—acknowledging differing responsibilities’

In this critical case, we discuss how the responsibilities formally recorded for PPI advisory roles foreground the distinctly different responsibilities and personal attributes required in differing PPI roles. We critique the research team's assumptions about advisory members’ capabilities to undertake multiple roles.

Informal involvement for the PPI person can be transformed into the more formal involvement if PPI members take on the role of co‐applicant: here PPI members share responsibilities with academic co‐applicants for governance and study delivery.^9^ The co‐applicant role implies significant contractual involvement over the lifetime of the research, contradicting expectations that PPI activity is solely and narrowly voluntary. Co‐applicants A and B both had experience of caring for a spouse with dementia and of PPI; their formal role in the applicant team brought tacit expectations they could take on active roles in the advisory groups. The differences in PPI advisory members’ and researchers’ assumptions about PPI roles and activity became evident in early PPI advisory group meetings. The research team were eager to discuss and progress an upcoming ethics application, but PPI advisory members, who were also co‐applicants, were more focused on gaining a clearer understanding of how the project was organized, staff hierarchies and where PPI roles fitted into these. This tension was managed by the (researcher) PPI coordinator consulting with advisory members to develop project organizational and activity planning sheets. The organizational charts were used to highlight where and how PPI activity would feed into management decisions. Forward planning activity sheets enabled advisory members to indicate their interest and availability to be involved in specific aspects of project activity such as developing study documents, reviewing data analysis outputs, discussing results and dissemination plans. In this project, the PPI advisory group shaped public‐facing documents such as newsletters and information sheets. They also identified specific research questions which expanded the research team's analyses. However during a review of PPI activity, we revisited advisory group minutes and realized we did not explicitly acknowledge by talk, or our practices, the formal and quasi‐legal roles of the PPI co‐applicants. By tracking and noting the absence of this topic, we can see that, if project governance challenges had arisen, our advisory members who were also co‐applicants might not have been adequately prepared by the researchers to actively recognize, report and act on such issues.

Understanding people's motivation to undertake PPI activity is important for them ensuring that personal needs are taken into account and met. Intrinsic reward is a lynchpin of volunteering. Although those undertaking PPI activity may get (limited) reimbursement for time and travel, for many it is a volunteer role they undertake from a desire to ‘give back’.^32^ PPI C explained that as an advisory member they had been encouraged to share knowledge and experiences, to question, explore, challenge and make suggestions, gaining rewards from knowing they could have impact on the research quality and content.

In our study, advisory members all expressed their commitment to the formal study aims. However their differing personal lived experiences as carers of people with advancing dementia informed their diverse opinions on how realistic it was to recognize people with dementia as ‘living independently’. This was evident when sharing qualitative results at an advisory meeting. Central to the research findings was seeing people living with dementia as agentic in maintaining their social participation. Discussing this finding brought extensive debate about whether the results were credible. PPI members cited their lived experience as carers to challenge the researchers’ interpretations of qualitative data collected from people with a relatively recent diagnosis. Current experiences of caring in advancing dementia made it difficult for PPI C to accept researchers’ interpretations of findings as accurate or representative. PPI A, a carer whose spouse had died several years earlier and who had been involved in many other research projects, appeared to have more experience of, and trust in, the analysis process as authentic and as grounding interpretations in valid data. Having views challenged can be uncomfortable both for PPI members and researchers, as one advisory member asserted ‘there is a need to respect carers’ experience and view of a situation, as the person with dementia may not have full understanding of how much support they need to be involved in the ways they are’. This meeting developed a consensus on acknowledging the importance of the interview accounts as the reality of the participant with dementia, and on the need for the research team to more clearly define the social and medical context for their results to avoid overstating the well‐being of those with dementia.

Another way to make PPI roles more intrinsically rewarding is to ensure they provide personal development opportunities. During this study, PPI A came to hold three distinct roles, each eliciting distinct tacit and explicit expectations. The research team had to consider both ethical and methodological issues to support this person's decision to undertake multiple roles. PPI A was already a co‐applicant and advisory member with lengthy PPI experience, when they expressed interest in co‐researching in interviewing, seeing this new role as a means to refine and further develop research skills. The research team were concerned there could be role responsibility conflicts. If the advisory member was collecting data, could they also maintain their co‐applicant and advisory role responsibilities? What ethical and analytic issues might arise from sharing their interview data at an advisor meeting?

Researchers voiced assumptions about PPI colleagues’ academic and personal abilities, questioning PPI members’ potential for reflexivity in their practice and their personal capacities to increase their PPI activity. Yet, the researchers themselves already took on such multiple roles, so contradicting the necessary logic of denying comparable support for PPI multiple roles within co‐research and co‐production. Here support meant ensuring both groups were clear about roles and responsibilities. The research team listened to and supported advisory members’ decisions to drop, or take‐up, any role available to them. In this case, our PPI colleague decided to manage their other responsibilities by not attending the advisory team meeting, while in co‐researcher role.

### 2.2 Case 2: ‘My experiences matter’—the complexity of co‐research interviewing’

Co‐research is a distinct form of PPI activity where people with lived experience of the condition contribute to research activities alongside professional researchers.^10^ Co‐research may improve the interview experience for participants and empower the co‐researchers by providing them with new skills, amplifying their voice and presence in the research process. However, our stance here is not to accept this view as an unproblematized given, but to actively examine potential areas of pragmatic and theoretical challenge within this PPI activity. This critical case explores the ethical status of the role, conflict in roles occasionally experienced by the co‐researchers and the challenges when multiple people contribute to an interview.

The support required for co‐research resembles those of all PPI activity; they require practical support and resources including training, transport and remuneration.^13^However, the ethical and governance demands of this activity may be more extensive and tighter than in other PPI activities that do not entail contact with research participants. We stated our intention to work with people with dementia and carers as co‐researchers in the ethics submission. In turn, the ethical review body required that co‐researchers be informed and protected in the same ways as research participants, namely provided with tailored information and consent forms. These were collaboratively developed with our PPI advisory committee. We question the equity of such ethical requirements as the role of PPI advisory member does not require ethical approval or governance oversight, even though these members might be asked to look at qualitative data and findings where they may be exposed to distressing data that strongly resonates with their own experience.

In co‐researching, people draw on their personal experiences of the conditions under study to guide and develop an interview. However in practice, our interview guides had already been ethically approved. This limited the scope of carer co‐researchers to direct the interview responsively. Importantly, to manage expectations it was made clear, to both co‐research and academic researchers, which aspects of the interview were negotiable and which were compulsory.

Qualitative interviewing often exposes the researcher to intense personal accounts. Such accounts may particularly resonate with a co‐researcher's own experiences of emotional anxiety and conflict in caring responsibilities. During our study, carer co‐researchers reported that at times they wanted to step away from the constraints of the interviewer role and offer advice to the people they were interviewing. The urge to alleviate participants’ anxiety raised specific tensions for carer co‐reseacher D, who struggled to balance the objective researcher stance with their experience of empathy as a fellow carer. This case raised moral and methodological questions. Morally, if any interviewer (not only a co‐researcher) holds information that might help a research participant in distress, they might be obliged to pass on this information, even if it impacts on project integrity.^33^ In this case, researcher‐carer‐co‐researcher debriefings enabled both to talk about differing interviewer roles in an interview situation. They agreed they could send a thank you letter to the research participant with generic information on support groups in the area, including one the carer co‐researcher ran. This provided information while leaving the participant free to take it up or not. Disclosing information during the interview might have changed the participant's current experiences before they shared it in the interview, or imposed obligations to follow‐up on contacts suggested.

Methodologically, the research team needed to rigorously consider whether co‐research activity would enhance data collection. Interviewing people in their own homes is complex, extensively debated in terms of the theoretical and moral concerns for interviewer‐interviewee power dynamics, researcher safety and presentation of self.^34^ A standard face‐to‐face interview offers an intimate space for researcher‐research participant conversation. In dementia research, the person with dementia and a family member more commonly take part together, and the researcher must manage conversations skillfully and responsively to foreground the account of the person with dementia.^18^

During our co‐researcher interviews, four people were often present. In most interviews, this did not impede interaction management: the carer co‐researcher led the interview, conversationally; the researcher pursued any points relevant to the study, and where necessary, the carer promoted and reassured the person with dementia about participating. However, in one case, a friend of the person with dementia was present and it was the carer co‐researcher's first interview, so nuanced ways of working had not yet developed between co‐researcher and researcher. From the start, the hearing and sight sensory loss of the participant (with dementia) impeded communication especially when several people spoke at once. Furthermore, the friend's and participant's accounts were frequently contradictory and the carer co‐researcher tended to refer back to the friend's account, affecting data quality. This suggested co‐research training should include clear guidance on tactics to sustain research focus on the main research participant's experience and voice. The researcher's reflective summary of this interview reported their desire to intervene to ‘get the interview back on track’. The researcher, where possible, re‐addressed questions answered by the friend, directly back to the research participant, maintaining the researcher's academic and ethical instinct to maintain ultimate control in data collection, while also empowering the voice of the person with dementia.

We involved the carer co‐researchers during the analysis phase, sharing interview transcripts and then discussing their interpretations of the data. Co‐researchers and the research team broadly agreed on the meaning of the data and the main themes. There were no difficulties in supporting carer co‐researchers to access transcribed data, yet this was a challenge when sharing data with people with dementia.

### 2.3 Case 3: ‘Co‐research or validation—doing data analysis with people with dementia?’

Consistent with the ethos of The PRIDE Study, and evident in our data, was recognizing that many people with dementia are keen to be involved in meaningful activity. PPI enables them to draw on their lived experience to inform research. After data collection, the research team tried further to involve people with dementia in PPI activity, this time in data analysis. To increase contact with people with dementia likely to have cognitive and physical capacity to undertake this more limited co‐researcher role, we collaborated with NHS research staff. Our earlier ethical approval proved invaluable, as it authorized NHS staff to pass on study information to people known to them as patients. The Alzheimer's Society also shared information with their research advisory group. After recruiting in two geographical locations, eight people with dementia agreed to be involved in the co‐researcher data analysis. We ran two consecutive half‐day workshops with four people at each location. The PPI coordinator, supported by NHS research staff and another qualitative researcher, facilitated each workshop and sustained the orientation of the person with dementia to the co‐research activity.

As researchers, we had to consider how to select and present qualitative data so people with mild to moderate dementia, without research experience, could relate to it. The analysis focus was informed by key topics relevant to The PRIDE study intervention manual. Varying presentation methods helped make qualitative interview data more accessible to lay people: data extracts were presented as either single sentence quotes, or half‐page case studies or short phrases. Each workshop provided four short, focused activities. In two activities, co‐researchers discussed the meaning of single sentences, thereby undertaking interpretation and developing codes. In a further ‘interpretation’ activity, co‐researchers considered the most important aspects of the short case studies. Finally, a ‘coding’ activity involved selecting a theme that best related to a short phrase

Our theoretical concern was whether this form of PPI activity constituted PPI validation or co‐research analysis. Validating the fit of results with the views of those living with the condition or those who have taken part in the research is commonly used to enhance credibility of results in qualitative research.^35^Here we argue, we moved beyond such validation to co‐research analysis as the co‐researchers could not only relate to the experiences of the research participants (a form of validation) but also recognize common experiences within the data, extend interpretations and compare or contrast data items. Table 1 illustrates examples of how this activity then shaped our wider on‐going analysis.

**Table 1:** Example of co‐researcher interpretations during data analysis with people with dementia

Extract from interview data	Co‐researcher extension and interpretation
‘The whole village is supportive if I was wandering round they would make sure I was alright’	*Confirmatory interpretation* ‘this is about feeling safe’
‘If you say you are losing your memory people's attitude change’	*Another analytical lens* ‘is there a gender difference as my female friends all seem to be embarrassed’
‘It's the small things which are a nuisance’	*Differing interpretations* The researcher has focused on the term ‘nuisance’ but the co‐researchers agreed that the most important part of the quote was the ‘small things’

To fit with this as a voluntary role, we needed to know how the co‐researchers living with dementia experienced the activity. They stated they enjoyed the activity and ‘could have more of this type of thing’ as ‘it's good to get together and talk about things’ (researcher reflective notes). Nonetheless, the resources need to organize this type of activity may be beyond a ‘usual’ PPI budget. The activity also raised specific ethical challenges. Throughout the sessions, we had to refocus the co‐researchers with dementia onto the task, by reminding them they were not research participants but were working with the research team to make sense of the data. Maintaining confidentiality also proved important as the activity generated much talk of personal experiences, as is common in PPI activity.

## 3 DISCUSSION

By embedding distinct PPI roles and activities within a programme grant, we could explore, understand and modify PPI activity to elicit and address tacit and explicit expectations of research teams’ and PPI members’ activities. We found PPI members could undertake multiple PPI roles. By addressing and adjusting the dynamics of situations, we could enable conflicting voices to be heard, so improving and making more transparent how we disseminated results. The role of co‐researcher entailed emotional work when personal experiences were brought to the fore in an interview context. We found research practices, if ‘conservative’, cannot support drawing on co‐researchers’ experiences to benefit the research participant. With careful planning and appropriately presenting qualitative data, people with dementia can be meaningfully involved with qualitative data analysis.

We found it important to make explicit and then address diverse views of PPI colleagues and researchers around roles, meetings activities and research impact. Renedo et al.^4^ reported PPI members actively referring to organizational cultures and structures to infer what people expected them to do. In our study, several PPI carer colleagues had been previously involved in other research projects and service improvement initiatives. They drew on these experiences to understand what the PRIDE Study might involve for them. This unsurprisingly led to their expressing diverse agendas in initial PPI advisory group meetings. Having shared expectations about PPI roles and each party expressing their values can help PPI process and outcomes.^36^ This highlights the responsibility of academic researchers to manage and guide PPI processes, while still ensuring equality of power and autonomy through recognizing individual accounts. We strived to create spaces and activities where PPI accounts could shape research outcomes; however, PPI impacts were small and locally located within this study. Green^37^ discusses the continuing challenge of increasing the impact the public can make in scientific research communities, when the biomedical model continues to dominate research decision making. This might be more so in dementia research where historically the voice of the person with dementia has been silenced. Nonetheless, there is growing recognition of the value of PPI with people with dementia.^38^

PPI members may not, and need not necessarily, represent all the experiences of the wider population impacted by the research project. Rather, PPI knowledge needs to be understood as that of a ‘specific insider’ who has access to ‘insider experience’ which may not be readily available either to the other research team members or the wider public. This was brought to the fore in discussions with carer PPI advisory members when there was discordance between the views of experienced carer PPI members and researchers when interpreting qualitative data. Frankham^39^argues that those on the ‘inside’ of an issue have ‘a different epistemology (way of knowing, understanding, experiencing the world) and that this needs to be taken into account *throughout* [our emphasis] the research process’ (2009:5). We suggest that ‘different ways of knowing’ within and between PPI and other researcher groups are rarely acknowledged when reporting PPI activity. It is rarer still to report ways in which any such different understandings are managed and/or resolved. If this is not made explicit, neither will there be transparency about who may have had the most powerful voice in deciding what is eventually reported and published. GRIPP guidelines on reporting PPI provide a framework for highlighting these theoretical dilemmas but they are not, as yet, widely used.^26^

Co‐research is an emerging method of working, particularly in dementia research.^40^, ^41^, ^42^, ^43^ We found it was a complex social activity where people needed to enact different roles in different situations. Unproblematically assuming that involving lay people in research activity will improve the participant experience, or the quality of the data, does not acknowledge the complexity of qualitative research encounters and the participatory and interpretive demands they bring. Researchers often undertake extensive training to enable them to develop and use reflexive interview skills to understand the techniques of probing and reiterating information to extend the data. While co‐researchers receive training, the aim is not to make them ‘expert’ researchers, so there remains a need to make explicit to everyone involved what the co‐researcher role and activities can be and how planned activities might support future knowledge claims.

In our study, carer co‐researchers experienced conflict between their desire to support others, which may have prompted their engagement in PPI, and their appreciation that researchers needed to be objective. While we addressed this retrospectively, providing post‐interview support to the co‐researcher, we suggest that the emotional work of co‐research is often not made as explicit as it needs to be, to both provide appropriate support in research activities and for understanding the analytic outcomes. Yet, the empathy that carer co‐researchers displayed towards participants appeared to enhance the rapport between interviewer and participant. There is some evidence that the empathetic co‐researcher can be a valuable resource in reducing participant distress.^44^To enable this specific PPI activity to develop in ways that can enhance and validate the experience for all participants, and the quality of data then produced, there is a need for further empirical understanding of what the co‐researcher role entails within different research contexts.

A novelty in this project was the opportunity to work with people with dementia in data analysis. When the study was commissioned in 2014, it was rarer for the voice of people with dementia to be heard outside the role of participant. There is now a significant, appropriate and growing presence of people with dementia who help shape research.^15^, ^38^ However, there are practical and epistemological challenges of involving people with dementia in co‐research activity.^40^, ^41^, ^42^, ^43^ Involving people with dementia in co‐research is an area where assumptions about capability and safety of people with dementia as needing protecting and lacking cognitive ability are still prominently reproduced by potential gatekeepers.^12^To address this in future work, we would more fully involve people with dementia who are already active within PPI activities to support our recruitment of other people with dementia to co‐researcher roles.

We found that during co‐researcher analysis activities, people with dementia were able to extend, and compare and contrast the data they encountered. As often found in PPI groups, activities included much talk around their personal experiences of living with dementia. Those who organize PPI activity may expect that a PPI member needs to justify their place within a PPI group by virtue of their representative or connected experiences. Researchers often comment on this requirement, sometimes stating it as unreasonable that PPI members need to recount their experience as justifying their ‘qualification’ for being in any PPI role. However, such interrogative social activity is perhaps no different from the induction practices of researchers and academics who, in new work situations, introduce and posit their right to be there by framing who they are so as to position and align their job role and expertise in the current work context. We found that co‐research involving people with dementia enabled them to see the value of their experiences and to gain personal satisfaction from being part of research. This builds on the work of Bartlett^45^ who reported the personal benefits of being actively involved in such citizenship activities. There is a need to explore this field further to clarify whether and how the positive satisfaction people report from co‐research activities is similar or different to that reported from ‘being a research participant’.

We acknowledge that our discussions and conclusions emerging through these PPI processes are based on experiences with a relatively small number of PPI colleagues and that we did not set out to undertake a formal evaluation of the PPI activity. However, the value of acknowledging and reflecting on such distinct PPI activities is the ability to recognize what knowledge is generated in what activities. Reflecting on social contexts and processes can improve practice throughout the study and in future work. The usefulness of the careful descriptions offered and review of the types of activities generated extends our awareness of the construction of PPI in practice and how this may be considered in other context. The findings of this work resonate with Mockford et al.^40^ who reported that overall the benefits to PPI members of greater skills and confidence and the benefit to the research of more substantial links with third sector partner organizations outweighed the costs and limits of challenges posed by having to negotiate organizational procedures such as research passports.

### 3.1 Implications for future PPI work

The key implications of these findings are to make transparent those factors that might make some PPI roles less accessibility and acceptability to some groups of people. For example, carrying out the quasi‐legal responsibilities inherent in the PPI co‐applicant role and the reliance of access to ICT may indeed make this role inaccessible to some people. This issue of access is of specific concern, and takes particular forms, in dementia research. The complex language and procedures commonly used to structure and regulate the PPI roles potentially exclude experiences from those with dementia.

PPI activity involving people with dementia may require specific preparation to ensure accessibility of materials, activities and venues. Such preparation may require additional resources (time and financial) when planning research. Nonetheless, the potential benefits of including the experience of those directly living with a dementia diagnosis and ensuring they can also access the personal gains commonly reported for PPI members indicate that this is a form of PPI activity worth pursuing for participants and for the research enterprise.

## 4 CONCLUSION

Reviewing and evaluating the diverse PPI roles and activities undertaken throughout a five‐year programme of research [xxx study] has enabled those involved to gain a nuanced understanding of the challenge of meaningfully embedding PPI in dementia research, specifically including people with dementia. PPI was presented here as a complex social activity that, therefore, challenged lay people to undertake multiple roles, each with differing responsibilities and accountability. Co‐research work is often resource‐heavy and needs to be fully accounted for in planning and implementing the research design. Co‐researchers, by drawing on their lived experiences, can bring new insights to research data and analysis, but there may be an emotional cost to this voluntary work. Responsibilities lie across the research team in resolving interpretive challenges which are raised when bringing greater diversity to research teams and activities.

## DATA SHARING

Data sharing is not applicable to this article as no new data were created or analysed in this study.
